# Editorial Comment: Larger patients shouldn’t have fewer options: urethroplasty is safe in the obese

**DOI:** 10.1590/S1677-5538.IBJU.2019.0511.1

**Published:** 2020-09-02

**Authors:** Luciano A. Favorito

**Affiliations:** 1 Unidade de Pesquisa Urogenital Universidade do Estado do Rio de Janeiro RJ Brasil Unidade de Pesquisa Urogenital, Universidade do Estado do Rio de Janeiro, Uerj, RJ, Brasil

## COMMENT

The present paper of Alger and colls ( 1 ) is very interesting and shows a very important topic in reconstructive urology: The impact of obesity in urethroplasty outcumes. Urethral surgery has more technical difficult in patients with high body mass index (BMI) ( 2 ). There are very factors implied in postoperative success of urethral surgery ( 3 - 5 ) but the BMI is one of the most studied ( 6 ). In this elegant paper the authors reviewed 193 patients with anterior urethral strictures who had undergone anastomotic or augmentation urethroplasty with at least 12 months follow-up and shows that despite the association with increased urethral stricture length and estimated blood loss, obesity is not predictive of adverse perioperative outcomes or stricture recurrence. Obese patients should be offered urethral reconstruction, but patient selection and preoperative counseling remain imperative.
